# A Rare Case of Hepatic Vanishing Bile Duct Syndrome Occurring after Combination Therapy with Nivolumab and Cabozantinib in a Patient with Renal Carcinoma

**DOI:** 10.3390/diagnostics12020539

**Published:** 2022-02-19

**Authors:** Karim Gourari, Julien Catherine, Soizic Garaud, Joseph Kerger, Antonia Lepida, Aspasia Georgala, Fabienne Lebrun, Maria Gomez Galdon, Thierry Gil, Karen Willard-Gallo, Mireille Langouo Fontsa

**Affiliations:** 1 Department of Oncology, Institut Jules Bordet, Université Libre de Bruxelles, 1070 Brussels, Belgium; karim.gourari@bordet.be (K.G.); joseph.kerger@bordet.be (J.K.); aspasia.georgala@bordet.be (A.G.); fabienne.lebrun@bordet.be (F.L.); thierry.gil@bordet.be (T.G.); 2 Department of Internal Medicine, CUB Hôpital Erasme, Université Libre de Bruxelles, 1070 Brussels, Belgium; julien.catherine@ulb.be; 3 Institute for Medical Immunology, Université Libre de Bruxelles, 6041 Gosselies, Belgium; 4 Molecular Immunology Unit, Institut Jules Bordet, Université Libre de Bruxelles, 1070 Brussels, Belgium; soizic.garaud@borbet.be (S.G.); karen.willard-gallo@bordet.be (K.W.-G.); 5 Department of Gastroenterology, Hepatopancreatology and Digestive Oncology, CUB Hôpital Erasme, Université Libre de Bruxelles, 1070 Brussels, Belgium; antonia.lepida@bordet.be; 6 Department of Pathology, Institut Jules Bordet, Université Libre de Bruxelles, 1070 Brussels, Belgium; maria.gomezgaldon@bordet.be

**Keywords:** checkpoint inhibitors, immune related adverse events, cholestasis, severe ductopenia, vanishing bile duct syndrome

## Abstract

Tyrosine kinase inhibitors (TKIs) and immune checkpoint inhibitors (ICIs) significantly improve the outcomes of patients with advanced clear cell renal cell carcinoma (ccRCC); however, high-grade toxicities can occur, particularly during combination therapy. Herein, we report a patient with advanced metastatic ccRCC, who developed grade 4 cholestasis during combined therapy with nivolumab and cabozantinib. After the exclusion of common disorders associated with cholestasis and a failure of corticosteroids (CS), a liver biopsy was performed that demonstrated severe ductopenia. Consequently, a diagnosis of vanishing bile duct syndrome related to TKI and ICI administration was made, resulting in CS discontinuation and ursodeoxycholic acid administration. After a 7-month follow-up, liver tests had returned to normal values. Immunological studies revealed that our patient had developed robust T-cells and macrophages infiltrates in his lung metastasis, as well as in skin and liver tissues at the onset of toxicities. At the same time, peripheral blood immunophenotyping revealed significant changes in T-cell subsets, suggesting their potential role in the pathophysiology of the disease.

## 1. Introduction

During the last decade, targeted therapies, such as the new-generation tyrosine kinase inhibitors (TKIs) and immune checkpoint inhibitors (ICIs), have transformed the treatment of clear cell renal cell carcinoma (ccRCC) in the advanced stages of the disease [1,2,3,4,5]. Despite the excellent results observed with these drugs, a significant proportion of patients never benefits from treatment and most initial responders will ultimately develop progressive disease.

Another drawback is represented by the burden of added toxicity associated with a combination strategy. Regarding overlapping toxicities, all vital organs can be affected, including the liver. In clinical trials, hepatitis was observed in up to 50% of patients during TKI treatment, but was generally transient and low-grade, self-resolving without treatment discontinuation [6]. Severe hepatotoxicity also varies according to the VEFGR-TKI molecules (e.g., pazopanib, sunitinib) [6]. The management of such toxicities is often difficult, partially owing to a poor understanding of their underlying pathogenesis, particularly in the setting of dual therapy due to the overlapping spectra of toxicity [6,7]. Safety data related to the concomitant use of ICIs and TKIs are issued from early phases of clinical trials or case reports, mostly corresponding to manageable toxicities which resolved after treatment discontinuation and/or administration of low dose corticosteroids (CS) [8].

Herein, we report a rare case of vanishing bile duct syndrome (VBDS) that arose after concomitant treatment with cabozantinib and nivolumab in the setting of an advanced metastatic ccRCC (mRCC). A detailed assessment of the peripheral blood and tissue immune responses is then presented.

## 2. Case Report

A 58-year-old man with a history of mRCC was hospitalized in the setting of a pruritic cutaneous rash, grade IV cholestasis (total bilirubin: 28 mg/dL, including 25 mg/dL conjugated; alkaline phosphatase: 805 UI/L), and grade IV hepatitis (aspartate amino-transferase: 694 UI/L; alanine amino-transferase: 1137 UI/L). This occurred one month after the introduction of a TKI inhibitor (cabozantinib, 40 mg/day) to his current treatment with nivolumab (3 mg/kg/3 weeks) started in the context of metastatic progression 8 months before. There were no recent medication changes, except the introduction of cabozantinib. Physical examination revealed a diffuse maculopapular skin eruption and conjunctival icterus, without any other sign of hepatic failure.

**Figure 1:**
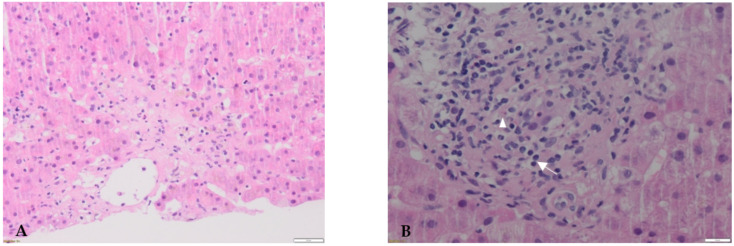
First liver biopsy performed at presentation. Hematoxylin and eosin-stained sections. (**A**): Liver parenchyma is characterized by centrilobular necrosis, sometimes confluent. (**B**): Immune infiltrate predominates around portal areas and is mainly composed of lymphocytes (arrow) and rare eosinophils (arrowhead).

Blood analyses rapidly ruled out infectious, metabolic, and autoimmune diseases. Ultrasound imaging and liver MRI excluded macroscopic hepatic metastasis and obstructive biliary disease. A first liver biopsy was performed and showed images of centrilobular necrosis and cholangitis with poor mixed inflammation consisting of eosinophils and lymphocytes, consistent with a drug reaction (Figure 1A,B). According to the presumed ICI-related adverse event (irAE) and/or TKI toxicity, nivolumab and cabozantinib were discontinued, and high-dose CS were started (methylprednisolone, 2 mg/kg/d). Seven days later, no improvement in serum bilirubinaemia was observed, prompting the addition of oral mycophenolate mofetil (MMF, 1 g/d) to CS according to ESMO guidelines [9]. Four weeks after dual immunosuppressive therapy (IT), following the lack of improvement in liver tests, CS were tapered to minimize side effects and a second biopsy was performed.

**Figure 2:**
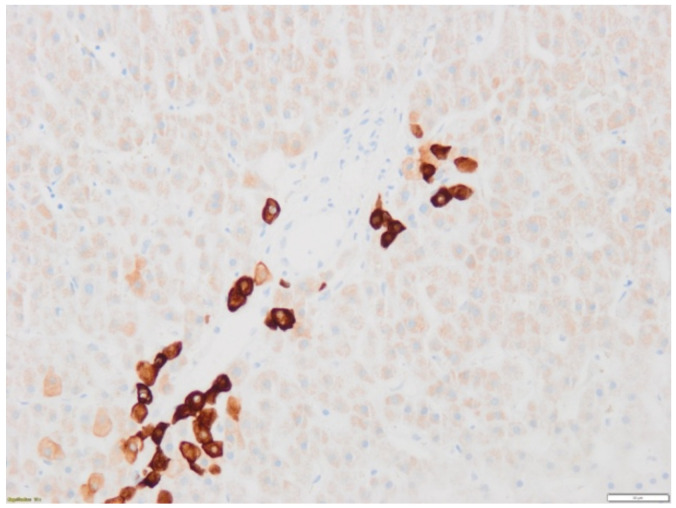
Second liver biopsy performed 5 weeks after cholestasis onset. Immunohistochemical staining with an anti-cytokeratin-7 (CK7) antibody. Large interlobular bile ducts are absent as anti-CK7 staining only reveals the ductular reaction. These findings are suggestive of ductopenia.

Remarkably, this second biopsy revealed a severe ductopenia with limited immune infiltrate within liver parenchyma, suggesting vanishing bile duct syndrome diagnosis (Figure 2). Accordingly, immunosuppressors were slowly tapered and ursodeoxycholic acid (UDCA) was given (5 mg/kg/d, with a progressive increase to 15 mg/kg/d). After 3 months, a slow decrease in serum conjugated bilirubin levels was observed until its normalization at 7 months. Considering the outcome of the patient, the time for a subsequent line for systemic progression was 8 months. Two years after the VBDS occurred, the patient is still alive.

## 3. Discussion

There has been significant progress in the treatment of patients with mRCC, especially with the recent approval of TKI and immunotherapy combination as a first-line treatment. In 2019, the FDA approved pembrolizumab (anti-PD-1) plus axitinib therapy, demonstrating survival advantages compared to sunitinib [10]. Since then, the combination of avelumab (anti-PD-L1) with axitinib has also been compared to sunitinib and finally approved (survival data are still immature; follow-up for the final analysis is ongoing) [11]. Despite their efficacy, the major concerns associated with these dual therapies are the potential cumulated and/or overlapping toxicities.

### 3.1. Case

Here, we report an exceptional hepatic manifestation, namely a vanishing bile duct syndrome, in a patient receiving cabozantinib and nivolumab for metastatic progression of a mRCC. VBDS is a rare condition in which patients develop chronic cholestasis associated with ductopenia, defined as a loss of interlobular bile ducts in >50% of portal areas following histological examination [12]. Causes of ductopenia are diverse and include drugs (e.g., antibiotics, anticonvulsants), immune disorders (e.g., primary biliary cholangitis), infectious diseases (EBV, CMV), and haematological malignancies (Hodgkin lymphoma) [12]. To our knowledge, this case represents the first VBDS occurring during VEGFR-TKI/ICI combination to date; however, Zhong and al. reported the onset of a fatal VBDS 25 days after pazopanib introduction, directly succeeding pembrolizumab therapy [13]. In our case, the recent initiation of cabozantinib suggests its potential causative role, despite the fact that grade IV cholestasis seems rare and can often be explained by other factors during VEGFR-TKI therapy (e.g., hepatic insufficiency associated with hepatocellular carcinoma) [6,14,15].

On the other hand, ICIs have been associated with various types of liver impairment, including marked cholestatic hepatitis with recent case reports describing steroid and MMF therapy-resistant disease where the biliary tract seemed the major target of injury, even if hepatocellular damages were also present [16,17]. Moreover, three cases of VBDS occurred in patients receiving ICIs alone, while no reports of TKI-related VBDS could be retrieved from the literature [7,18,19]. Table 1 summarizes the main clinical features of those patients. Briefly, VBDS was always associated with pembrolizumab use, only appeared after an injection in two cases, and induced a mean elevation of conjugated bilirubinemia of 26.3 mg/dL. ICIs were discontinued in all patients, and CS, MMF, and UDCA were used in two cases while the remaining patient declined any therapy. Surprisingly, this last patient normalized his hepatic tests after 16 weeks, while another required CS and UDCA to achieve the same outcome. The third patient was ultimately treated by plasmapheresis and died 26 days after cholestasis onset. These observations, including ours, suggest that ductal loss leading to VBDS helps to explain why IT could be ineffective once ductal damages appear. In our case, whether cabozantinib had enhanced the immune response especially towards ductal tissue or impaired cellular regeneration after immune destruction is unknown; however, this could explain the timescale between VEGFR-TKI initiation and cholestasis occurrence.

### 3.2. Biomarkers

Given the unique features of this case and the absence of any relevant pathophysiologic mechanism underlying VBDS during ICI/VEGFR-TKI therapy, we decided to assess local immune responses occurring in our patient at the onset of adverse events. First, we studied tissular immune markers in lung metastasis at disease progression, and in skin and liver tissues at the onset of irAEs using multiplex immunohistochemistry (mIHC) on formalin-fixed and paraffin-embedded (FFPE) tissue from lung metastasis (January 2019), skin eruption (November 2019), and liver biopsies at the onset of the irAES (November 2019 and January 2020). The methodological approach related to tissue immunophenotyping using mIHC is presented in Supplementary Data S1.

**Figure 3:**
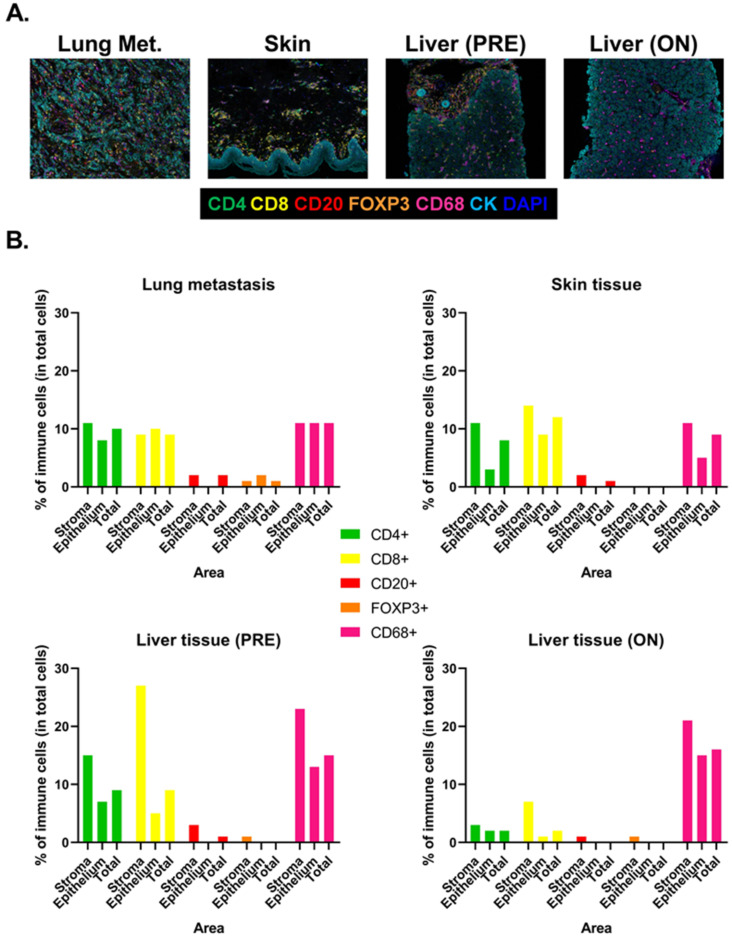
Tissue immune infiltration at disease progression and at the onset of toxicities. (**A**): Representative fluorescent multiplexed IHC images of lung metastasis, skin tissue, and liver tissues before (PRE) and during (ON) immunosuppressive therapy. Tumor-infiltrating lymphocytes were revealed by CD20 (red), CD8 (yellow) and CD4 (green), FOXP3 (blue), CD68 (magenta), and tumor cells by panCK (cyan). (**B**): Quantification of immune cell (as percentage of total cells) within the stroma, the epithelium, and total area of each tissue.

Liver biopsies were taken before (PRE) and during (ON) IT (CS and MMF) (Figure 3A). We measured the relative amount (percentage of total cells) of CD4+, CD8+ T cells, CD20+ B cells, FOXP3+ regulatory T cells (Treg), and CD68+ macrophages within the stroma and the epithelium area of each tissue. All tissues showed immune cell infiltration, including CD4+, CD8+ T cells, and macrophages, both in the stroma and epithelial areas (Figure 3B). B cells and Treg were less represented. Interestingly, T cell infiltration in liver was reduced by IT, including both CD4+ (15% versus 3% of total cells in the stroma) and CD8+ (27% versus 7% of total cells in the stroma) T cells. CD68+ macrophages were not impacted by IT in liver. These findings suggest that despite IT being able to significantly reduce lymphoid infiltration in the liver, their effects were not sufficient, either because ductal damages were already too important to allow a rapid recovery or because other cells were implicated in bile ducts’ destruction (e.g., macrophages).

**Figure 4:**
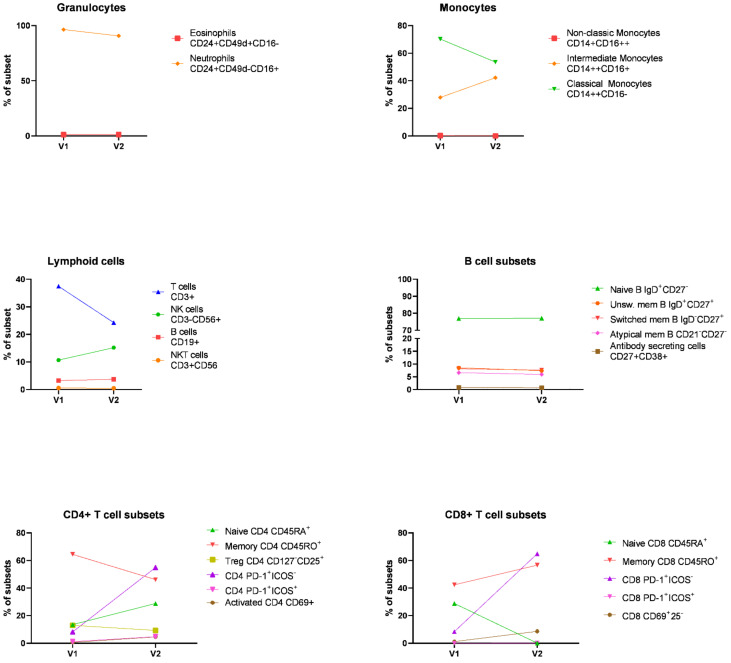
Peripheral blood immune cell profiling at the onset of toxicities. Immunophenotyping of granulocytes and mononuclear cells during immunosuppressive therapy at two timepoints after the onset of toxicities (visit 1 and 2) using flow cytometry.

We then evaluated immunologic changes in the peripheral blood on IT at two timepoints after the onset of toxicities (visit 1 and 2) using flow cytometry (Figure 4). The methodological approach relating to flow cytometry analyses is presented in Supplementary Data S2 and S3. First, we examined granulocytes and mononuclear cells, including monocytes and lymphocytes, in whole blood. We observed that frequency of classical monocytes, defined as CD14++CD16-, was reduced during treatment in combination with an increase in intermediate monocytes, defined as CD14++CD16+. Immunosuppressive therapy also reduced the frequency of T cells. A slight increase in NK cells was observed during the treatment. Peripheral blood mononuclear cells (PBMCs) immunophenotyping confirmed the reduction in T cells, including both CD4+ and CD8+ subsets, and a slight increase in B cells during treatment (Figure 4). Activated T cells, defined as CD69+ cells, were not present at visit 1 (less than 1% of T cells); however, the frequency of CD69+CD4+ and CD69+CD8+ T cells increased during IT (7% and 12%, respectively). We also quantified PD-1 expression among CD4+ and CD8+ T cells. PD-1+CD4+ and CD8+ T cells represented 8% of T cells at the onset of toxicities and their frequency substantially increased during therapy (55% and 65%, respectively). These PD-1+ T cells did not express ICOS, i.e., an activation marker. Immunosuppressive therapy (CS and MMF) also impacted T cell maturation but in a different way for CD4+ and CD8+ T cells. The frequency of memory CD8+ T cells increased during treatment while the frequency of memory CD4+ decreased.

In line with these observations, robust T-cell infiltration, activation, and clonal expansion were already described in the cardiac and skeletal muscle of two patients with melanoma who developed fatal myocarditis after treatment with ipilimumab and nivolumab [20]. Further studies of T cells reactivity within tumour microenvironment are needed to elucidate their contribution in toxicities.

Metastatic ccRCC patients treated with nivolumab from the CheckMate-010 trial revealed that a high percentage of CD8+ tumour-infiltrating lymphocytes (TIL) that are PD-1+TIM-3-LAG-3- within the tumour microenvironment (assessed by mIHC) correlated with high levels of T-cell activation, including cytolytic activity and IFNγ response, and was associated with longer median immune-related PFS and a higher odds ratio rate on nivolumab [21]. However, CyTOF analyses on clinical samples from melanoma patients treated with ICIs did not show any association between the frequency of PD-1 expression on either CD4+ or CD8+ T cells with clinical benefit [22]. In the current study, the presence of PD-1+ T cells was not assessed by mIHC; however, an increase in PD-1+ CD4+ and CD8+ T cell frequencies was observed in the peripheral blood on IT at the onset of toxicities. Further analyses are required to understand whether the expression of PD-1 reflects T cell exhaustion/dysfunction or activation of anti-tumour response, and whether these cells could be responsible for hepatic duct injury in this setting of ICI/TKI-induced VBDS.

## 4. Conclusions

Given overlapping hepatic toxicities of TKIs and ICIs, severe liver impairment can occur with a delayed management because of the poor understanding of the underlying pathogenesis. Although data concerning toxicity and management of each drug used as monotherapy are becoming increasingly available, the current lack of knowledge regarding potential toxicity of their combined administration should prompt thorough evaluation in relation to liver biopsy, case reporting, and translational research. In particular, the occurrence of immunosuppressor-resistant cholestatic hepatitis should raise suspicion for vanishing bile duct syndrome in patients treated with TKI/ICI bi-therapy.

## Figures and Tables

**Table 1:** Published cases of vanishing bile duct syndromes occurring during immune checkpoint inhibitor and/or tyrosine kinase inhibitor therapy.

Reference	Neoplasia	Suspected Causal Therapy	Time-to-Onset	Peak c- Bilirubin/ALAT	Signs of Hepatic Failure	Therapy	Issue
Doherty, 2017 [7]	Melanoma	Pembrolizumab	1 cycle (8 d)	23 mg/dL 1536 IU/L	Absent	CS 1 m/kg, MMF, UDCA	Normalized c-bili under CS and UDCA therapy
Thorsteinsdottir, 2020 [18]	Melanoma	Pembrolizumab	12 cycles	32 mg/dL 450 IU/L	Absent	CS 2 mg/kg MMF, UDCA, plasmapheresis	Death (d+26 after cholestasis onset)
Zhong, 2020 [13]	mRCC	Pembrolizumab (previously) Pazopanib (ongoing)	5 cycles (25 d)	22 mg/dL 800 IU/L	Absent	CS 2 mg/kg, UDCA	Death (d+30 after cholestasis onset)
Masetti, 2021 [19]	NSCLC	Pembrolizumab	1 cycle (21 d)	24 mg/dL 818 IU/L	Absent	None (patient refusal)	Normalized c-bili after 16 weeks
Present case	mRCC	Nivolumab, Cabozantinib (both ongoing)	6 cycles (28 d)	28 mg/dL 1137 IU/L	Absent	CS 2 mg/kg, MMF, UDCA	Normalized c-bili after 28 weeks under UDCA therapy

Abbreviations: ALAT, alanine aminotransferase; c-bili, conjugated bilirubin; CS, corticosteroids; d, day; IU, international unit; MMF, mycophenolate mofetil; mRCC, metastatic renal cell carcinoma; NSCLC, non-small cell lung carcinoma; UDCA, ursodeoxycholic acid.

## Data Availability

The data presented in this study are available on request from the corresponding author. The data are not publicly available due to the very limited amount of unpublished data generated during this study.

## Supplementary Materials

The following supporting information can be downloaded at: www.mdpi.com/article/10.3390/diagnostics12020539/s1, Data S1: Tissue immunophenotyping; Data S2: Peripheral blood immunophenotyping; Data S3: Table S1: Antibodies used for flow cytometry.
