# Supplementary material for: A Rare Case of Hepatic Vanishing Bile Duct Syndrome Occurring after Combination Therapy with Nivolumab and Cabozantinib in a Patient with Renal Carcinoma
Source: Diagnostics (Basel). 2022 Feb 19;12(2):539. doi: 10.3390/diagnostics12020539 (PMC8871391; doi:10.3390/diagnostics12020539)
Supplement: Supplementary file 1 [file diagnostics-12-00539-s001.zip › diagnostics-1582214-SI.pdf]

Supplementary Data.

Data S1. Tissue immunophenotyping.

Four-micrometer sections from full FFPE blocks were used for manual multiplex immunohistochemistry (mIHC) staining according to the manufacturer's instructions (Opal 7 Solid Tumor Immunology kit, Akoya Biosciences®).
The used antibodies were CD4 (80 ng/ml, 90 min, Akoya Biosciences®), CD8 (68 ng/ml, 90 min, Akoya Biosciences®), CD20 (45 ng/ml, 90 min, Akoya Biosciences®), FOXP3 (674 ng/ml, 90 min, Akoya Biosciences®), CD68 (16 ng/ml, 90 min, Akoya Biosciences®), and panCK (400 ng/ml, 90 min, Akoya Biosciences®).
Slides were mounted with Vectashield Hardset Antifade Mounting Medium (VectorLaboratories). Multiplexed slides were scanned on Vectra Polaris Automated Quantitative Pathology Imaging System (Akoya Biosciences®). InForm® Tissue Finder™ software was used in order to deconvolute the multispectral images, to segment issue, and to segment and phenotype cells. The percentage of cells was calculated on full section except for lung metastasis for which 25 % of the section was multispectrally acquired. Digital quantification was then performed using PhenoptrReports (Akoya Biosciences®). GraphPad Prism8 software was used to graph individual datapoint.

Data S2. Peripheral blood immunophenotyping.
Fifty-microliter of whole blood pre-treated with Human Fc Receptor Binding Inhibitor (eBiosciences) was incubated with manufacturer's suggested dilutions of fluorescently labelled primary monoclonal antibodies (*Supplementary Table 1*) for 30 min at 4°C followed by red blood cell lysis buffer (Miltenyi Biotec) during 10 min at room temperature.
Peripheral blood mononuclear cells (PBMC) were purified by density gradient centrifugation over Lymphoprep™ (Stemcell technologies) and washed three times before flow cytometry staining. PBMC were incubated with manufacturer's suggested dilutions of fluorescently labelled primary monoclonal antibodies (Supplementary Table 1) for 30 min at 4°C followed by washing with of PBS. Whole blood and PBMC were then immediately acquired on a GALLIOS 10/3 cytometer (Beckman Coulter), and analysed on Kaluza Flow Cytometry Analysis v1.2 software (Beckman Coulter).

Data S3. Supplementary Table S1. Antibodies used for flow cytometry.

Name	Conjugaison	Firme	Applications
CCR7	APC/Cy7	BioLegend	PBMC
CD103	PE	Miltenyi	PBMC
CD127	APC	eBiosciences	PBMC
CD138	APC	Miltenyi	PBMC
CD14	APC-Vio770	Miltenyi	Whole Blood
CD15	eFluor450	eBiosciences	Whole Blood
CD16	PC7	eBiosciences	Whole Blood
CD19	APC-Vio770	Miltenyi	Whole Blood, PBMC
CD1d	PercPeF710	eBiosciences	PBMC
CD20	Alexa Fluor700	eBiosciences	PBMC
CD21	FITC	Miltenyi	PBMC
CD24	FITC	Miltenyi	Whole Blood
CD25	PercPeF710	eBiosciences	PBMC
CD27	PC7	Miltenyi	PBMC
CD3	eFluor450	eBiosciences	Whole Blood, PBMC
CD38	APC	Miltenyi	PBMC
CD38	PerCP eFluor710		PBMC
CD39	ViobrightFITC	Miltenyi	PBMC
CD4	AlexaFluor70	eBiosciences	Whole Blood, PBMC
CD44	VioBlue	Miltenyi	PBMC
CD45	Pacific Orange	eBiosciences	Whole Blood, PBMC
CD45RA	PE-Vio770	Miltenyi	PBMC
CD45RO	FITC	Becton Dickinson	PBMC
CD49d	APC	Miltenyi	Whole Blood

CD56	PE		Whole Blood
CD56	FITC	Miltenyi	PBMC
CD62L	PE-Vio770		PBMC
CD64	PercPeF710	eBiosciences	Whole Blood
CD69	PE	eBiosciences	Whole Blood, PBMC
CD8	PE-eFluor610	eBiosciences	Whole Blood, PBMC
ICOS	PC7	eBiosciences	PBMC
IgD	PE	Miltenyi	PBMC
IgG	VioBlue	Miltenyi	PBMC
PD-1	Viobright-FITC	Miltenyi	PBMC
